# A novel prediction approach using wavelet transform and grey multivariate convolution model

**DOI:** 10.1016/j.mex.2023.102259

**Published:** 2023-06-15

**Authors:** Flavian Emmanuel Sapnken, Marius Tony Kibong, Jean Gaston Tamba

**Affiliations:** a Laboratory of Technologies and Applied Science, IUT Douala, P.O. Box 8698, Douala, Cameroon; b Transports and Applied Logistics Laboratory, University Institute of Technology, University of Douala, P.O. Box 8698, Douala, Cameroon; c Energy Insight-Tomorrow Today, PO Box 2043, Douala, Cameroon

**Keywords:** Electricity forecasting, Grey systems, Wavelet transform, Convolution integrals, *Optimal discrete grey multivariate convolution model*

## Abstract

It is crucial to develop highly accurate forecasting techniques for electricity consumption in order to monitor and anticipate its evolution. In this work, a novel version of the discrete grey multivariate convolution model (ODGMC(1,N)) is proposed. A linear corrective term is included in the conventional GMC(1,N) structure, parameter estimation is carried out in a manner consistent with the modelling process, and an iterative technique is used to get the cumulated forecasting function of ODGMC(1,N). As a result, the forecasting capacity of ODGMC(1,N) is more reliable and its stability is enhanced. For validation purposes, ODGM(1,N) is applied to forecast Cameroon's annual electricity demand. The results show that the novel model scores 1.74% MAPE and 132.16 RMSE and is more precise than competing models.

• ODGMC(1,N) corrects the linear impact of t on the forecasting performance.
• Wavelet transform is used to remove irrelevant information from input data.
• The proposed model can be used to track annual electricity demand.

## Graphical abstract

**Figure:**
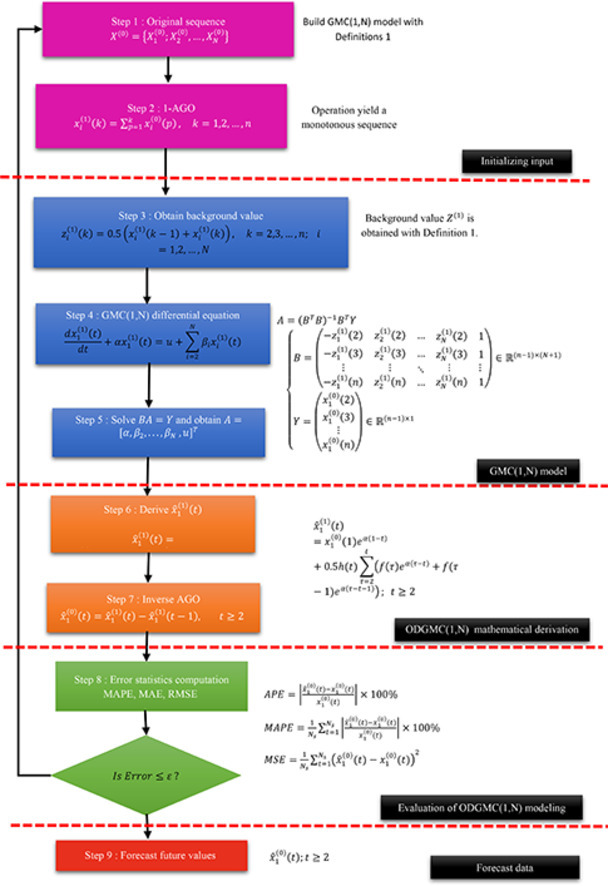

Specifications table

**Table:** 

Subject area:	Energy
more specific subject area:	*modelling and forecasting*
Name of your method:	*Optimal discrete grey multivariate convolution model*
Name and reference of original method:	B. Zeng, C. Luo, S. Liu, Y. Bai, C. Li, Development of an optimization method for the GM(1,N) model, Engineering Applications of Artificial Intelligence. 55 (2016) 353–362. 10.1016/j.engappai.2016.08.007. Y. Zhang, J. Zhang, L. Yu, Z. Pan, C. Feng, Y. Sun, F. Wang, A short-term wind energy hybrid optimal prediction system with denoising and novel error correction technique, Energy. 254 (2022) 124,378. 10.1016/j.energy.2022.124378.
Resource availability:	*World Bank statistics* (https://data.worldbank.org/) *International Energy Agency* (https://www.iea.org/) *Ministry of Energy* (http://www.minee.cm/)

## Introduction

It is essential to project future electricity needs since doing so lays the groundwork for improved utility-wide decisions [1]. The more accurate the forecasts, the better the decisions, which results in a utility that is more efficient and dependable and can better meet society's need for electric power. As a result, accurate forecasts are a tool for the grid's safe and dependable operation as well as for promoting cost-effective operation by optimizing production scheduling.

The legal forecasting requirement established by the Electricity Sector Regulatory Agency is one of the factors driving forecasting studies by electricity distribution system operators. According to the laws and network rules, information transfer to established contractual partners in the Central African and Cameroonian markets is required. Additionally, the regulations and related laws call for the payment of fairly substantial penalties against distributors in situations when they are the source of unbalanced electricity off-take from the grid. In these situations, distributors are compelled to provide forecasts, particularly during the dry season, when residential end-user usage might increase significantly because of the excessive heat conditions. The requirement for determining future investments in energy markets (including renewable energy sources), for enhancing energy efficiency, and for prioritizing energy investment projects also drives the development of electricity forecasting systems.

With unexpectedly high price spikes and daily seasonality, power price trends have peculiar characteristics. The need for accurate price estimates emerges as one of the fundamental challenges that the various market participants face [2], and as a result, research on electricity markets has taken centre stage in the electricity industry. Making money is the primary goal of these activities. It is crucial to keep in mind that the stability of the grid is the primary goal of the electricity markets [3]. However, the more stability of the system is threatened, the likelihood of a blackout increases as price volatility increases. Because of this, by accurately forecasting electricity consumption, which requires knowledge of future pricing, the grid's stability is could be improved.

The network operating margins must be in line with consumer demand and temporal variances for all of the aforementioned reasons. To some extent, operations must be adaptable in order to account for changes in demand. Predictive models developed for certain consumer categories, such as residential end users, are used to adapt power demand fluctuations with system operational restrictions. From the distributor's perspective, precise demand forecasting will lower operational expenses and remove penalties that can result from imbalances in supply and demand quantities.

## Methods and principles

### Defects of GMC(1,N) model

Assume that a system has N variables (or sequences) defined as follows:

(1) $$X^{\left(0\right)}=\left\{X_{1}^{\left(0\right)};X_{2}^{\left(0\right)},\ldots ,X_{N}^{\left(0\right)}\right\}$$

where $X_{1}^{\left(0\right)}$ represents the dependant variable. There are N−1 inputs in the system defined by $X_{i}^{\left(0\right)}\left(i\;=\;2,\;.\;.\;.,\;N\right)$ which represent explanatory variables (or independent variables). $X_{1}^{\left(0\right)}$ and $X_{i}^{\left(0\right)}\left(i\;=\;2,\;.\;.\;.,\;N\right)$ are strongly correlated with each other. We also assume that the sequence length for each variable $X_{i}^{\left(0\right)}\left(i\;=\;1,\;.\;.\;.,\;N\right)$ is n, so:

(2) $$X_{i}^{\left(0\right)}=\left\{x_{i}^{\left(0\right)}\left(1\right),x_{i}^{\left(0\right)}\left(2\right),\ldots ,x_{i}^{\left(0\right)}\left(n\right)\right\},\;i=1,2,\ldots ,N.$$

GMC(1,N) relies on the mean sequence and 1-AGO (the first order accumulating generation operator) similar to the classical GM(1,N) model. Eq. (3) is the 1-AGO sequences of $X_{i}^{\left(0\right)}\left(i\;=\;1,\;.\;.\;.,\;N\right)$:

(3) $$X_{i}^{\left(1\right)}=\left\{x_{i}^{\left(1\right)}\left(1\right),x_{i}^{\left(1\right)}\left(2\right),\ldots ,x_{i}^{\left(1\right)}\left(n\right)\right\},\;i=1,2,\ldots ,N.$$

where:

$$x_{i}^{\left(1\right)}\left(k\right)=\sum_{p=1}^{k}x_{i}^{\left(0\right)}\left(p\right),\;k=1,2,\ldots ,n$$

Eq. (4) represents the mean sequences $Z_{i}^{\left(1\right)}$ produced by consecutive terms of $X_{i}^{\left(1\right)}$:

(4) $$Z_{i}^{\left(1\right)}=\left\{z_{i}^{\left(1\right)}\left(1\right),z_{i}^{\left(1\right)}\left(2\right),\ldots ,z_{i}^{\left(1\right)}\left(n\right)\right\},\;i=1,2,\ldots ,N.$$

where:

$$z_{i}^{\left(1\right)}\left(k\right)=0.5\left(x_{i}^{\left(1\right)}\left(k-1\right)+x_{i}^{\left(1\right)}\left(k\right)\right),\;k=2,3,\ldots ,n;\;i=1,2,\ldots ,N$$

Definition 1 Let's consider the independent and dependant variable sequences to be $X_{i}^{\left(0\right)}\left(i\;=\;2,\;.\;.\;.,\;N\right)$ and $X_{1}^{\left(0\right)}$ respectively. With $X_{i}^{\left(1\right)}\left(i\;=\;1,\;.\;.\;.,\;N\right)$ as defined in Eq. (3), the GMC(1,N) model is then given by Eq. (5):

(5) $$\frac{{dx}_{1}^{\left(1\right)}\left(t\right)}{dt}+{\alpha x}_{1}^{\left(1\right)}\left(t\right)=u+\sum_{i=2}^{N}\beta_{i}x_{i}^{\left(1\right)}\left(t\right)$$

where u is the model parameter, $\sum_{i=2}^{N}\beta_{i}x_{i}^{\left(1\right)}\left(t\right)$ and $\beta_{i}$ are the deriving term and deriving coefficients respectively, while −α is referred to as the system development coefficient.

The differential equation (DE) model family includes GMC(1,N) as shown in Definition 1. The right-hand side (RHS) of Eq. (5) is generally not a constant, in contrast to the conventional GM(1,1). As a result, the GMC(1,N) model's time response function^1^ or solution to Eq. (5) is substantially more complicated than that of the GM(1,1) model. Ref. [4] first approximates Eq. (5) as a difference equation (Eq. (6b)) by considering the RHS of Eq. (5) to be a function f(t) as in Eq. (6a):

(6a) $$u+\sum_{i=2}^{N}\beta_{i}x_{i}^{\left(1\right)}\left(t\right)=f\left(t\right)$$

(6b) $$x_{1}^{\left(0\right)}\left(t\right)+{\alpha z}_{1}^{\left(1\right)}\left(t\right)=u+\sum_{i=2}^{N}\beta_{i}z_{i}^{\left(1\right)}\left(t\right),\;t=2,\ldots ,n$$

Eq. (6b) is obtained by integrating Eq. (5) on either sides in the range [t−1,t] and then applying the trapezoidal integration rule to the remaining unknown terms. In light of the parameters $A={\left[\alpha ,\;\beta_{2},\;.\;.\;.,\;\beta_{N},\;u\right]}^{T}$, the difference equation (Eq. (6b)) is a set of linear equations. Specifically:

(7) BA=Y

where:

(8) $$\left\{ \begin{array}{c} B=\left(\begin{array}{ccccc} -z_{1}^{\left(1\right)}\left(2\right) & z_{2}^{\left(1\right)}\left(2\right) & \ldots & z_{N}^{\left(1\right)}\left(2\right) & 1\\ -z_{1}^{\left(1\right)}\left(3\right) & z_{2}^{\left(1\right)}\left(3\right) & \ldots & z_{N}^{\left(1\right)}\left(3\right) & 1\\ \vdots & \vdots & \ddots & \vdots & \vdots \\ -z_{1}^{\left(1\right)}\left(n\right) & z_{2}^{\left(1\right)}\left(n\right) & \ldots & z_{N}^{\left(1\right)}\left(n\right) & 1 \end{array}\right)\in R^{\left(n-1\right)\;\times \;\left(N+1\right)}\\ Y=\left(\begin{array}{c} x_{1}^{\left(0\right)}\left(2\right)\\ x_{1}^{\left(0\right)}\left(3\right)\\ \vdots \\ x_{1}^{\left(0\right)}\left(n\right) \end{array}\right)\in R^{\left(n-1\right)\times \;1} \end{array}\right.$$

If $\det(B^{T}B)\neq 0$ (which implies that the matrix $B^{T}B$ can be inverted), then the parameters of matrix $A={\left[\alpha ,\;\beta_{2},\;.\;.\;.,\;\beta_{N},\;u\right]}^{T}$ can be estimated by least-squares method:

(9) $$A={\left(B^{T}B\right)}^{-1}B^{T}Y$$

Going further, Tien [4] solved the DE given in Eq. (5) using the initial condition (when t=1) ${\hat{x}}_{1}^{\left(1\right)}\left(1\right)=x_{1}^{\left(0\right)}\left(1\right)$, and obtained:

(10) $${\hat{x}}_{1}^{\left(1\right)}\left(t\right)=x_{1}^{\left(0\right)}\left(1\right)e^{\alpha \left(1-t\right)}+\int_{\tau =1}^{\tau =t}e^{\alpha \left(\tau -t\right)}f\left(\tau \right)d\tau$$

Eq. (10) is known as the cumulated forecast function of GMC(1,N). Ref. [4] provides a thorough explanation of how the solution was derived. However, it is still challenging to obtain an explicit expression because the convolution integral exists on the RHS of Eq. (10). Fortunately, we can get a rough solution by using some numerical integrals. The trapezoid formula is a straightforward and widely used technique that yields a more precise time response function shown below^2^:

(11) $${\hat{x}}_{1}^{\left(1\right)}\left(t\right)=x_{1}^{\left(0\right)}\left(1\right)e^{\alpha \left(1-t\right)}+0.5h\left(t\right)\sum_{\tau =2}^{t}\left(f\left(\tau \right)e^{\alpha \left(\tau -t\right)}+f\left(\tau -1\right)e^{\alpha \left(\tau -t-1\right)}\right);\;t\geq 2$$

The function h(t) is the step size given by:

$$h\left(t\right)=\left\{ \begin{array}{c} 0,\;t<2\\ 1,\;t\geq 2 \end{array}\right.$$

Finally, the forecasts ${\hat{x}}_{1}^{\left(0\right)}\left(t\right)$ are determined by applying inverse 1-AGO:

$${\hat{x}}_{1}^{\left(0\right)}\left(t\right)={\hat{x}}_{1}^{\left(1\right)}\left(t\right)-{\hat{x}}_{1}^{\left(1\right)}\left(t-1\right),\;t\geq 2$$

It is important to highlight that GMC(1,N) is an evolution of both the conventional GM(1,1) and GM(1,N) models. Also, we make the following key remarks:

Remark. 1 The second term in the RHS of Eq. (5) disappears if there is only one series (N=1, and is known as a univariate model) and the model is reduced to:

$$\frac{{dx}_{1}^{\left(1\right)}\left(t\right)}{dt}+{\alpha x}_{1}^{\left(1\right)}\left(t\right)=u,$$

This DE is the GM(1,1) model's traditional image equation. From Eq. (10), we obtain the following by setting f(τ) equal to a fixed value u:

$$x_{1}^{\left(1\right)}\left(1\right)=x_{1}^{\left(0\right)}\left(1\right)e^{\alpha \left(1-t\right)}+\int_{\tau =1}^{\tau =t}e^{\alpha \left(\tau -t\right)}ud\tau \;=x_{1}^{\left(0\right)}\left(1\right)e^{\alpha \left(1-t\right)}+\frac{u}{\alpha }\left(e^{\alpha t}-e^{\alpha }\right)e^{-\alpha t}=\frac{u}{\alpha }+\left(x_{1}^{\left(0\right)}-\frac{u}{\alpha }\right)e^{\alpha \left(1-t\right)}\;$$

which is the GM(1,1) model's time response function. The least-squares method of Eq. (7) can still be used to solve the parameters u and α. Except that B is reduced to:

(12) $$B=\left(\begin{array}{c} -\begin{array}{cc} z_{1}^{\left(0\right)}\left(2\right) & 1 \end{array}\\ \begin{array}{cc} -z_{1}^{\left(0\right)}\left(3\right) & 1 \end{array}\\ \begin{array}{c} \;\begin{array}{cc} \vdots \; & \;\vdots \end{array}\\ \begin{array}{cc} -z_{1}^{\left(0\right)}\left(n\right) & 1 \end{array} \end{array} \end{array}\right)\in R^{\left(n-1\right)\times \;2}$$

Remark 2 GMC(1,N) provided by Eq. (5) reduces to Eq. (13) if u=0.

(13) $$\frac{{dx}_{1}^{\left(1\right)}\left(t\right)}{dt}+{\alpha x}_{1}^{\left(1\right)}\left(t\right)=\sum_{i=2}^{N}\beta_{i}x_{i}^{\left(1\right)}\left(t\right)$$

Eq. (13) is the GM(1,N) model's standard image equation. The RHS of Eq. (13) is viewed as a constant in the pioneers’ works (see Ref. [5]). As a result, the cumulated forecast function of GM(1,N) may be derived exactly as that of the conventional GM(1,1):

$${\hat{x}}_{1}^{\left(1\right)}\left(1\right)=\left(x_{1}^{\left(0\right)}\left(t\right)-\frac{1}{\alpha }\sum_{i=2}^{N}\beta_{i}x_{i}^{\left(1\right)}\left(t\right)\right)e^{\alpha \left(t-1\right)}+\frac{1}{\alpha }\sum_{i=2}^{N}\beta_{i}x_{i}^{\left(1\right)}\left(t\right).$$

Compared to GMC(1,N), GM(1,N) contains one fewer parameter in the matrix $\tilde{A}={\left[\alpha ,\;\beta_{2},\;.\;.\;.,\;\beta_{N}\right]}^{T}$. The least-squares technique can also be used to calculate the values of these parameters. B also shows a difference, which is:

$$B=\left(\begin{array}{cccc} -z_{1}^{\left(1\right)}\left(2\right) & x_{2}^{\left(1\right)}\left(2\right) & \ldots & x_{N}^{\left(1\right)}\left(2\right)\\ -z_{1}^{\left(1\right)}\left(3\right) & x_{2}^{\left(1\right)}\left(3\right) & \ldots & x_{N}^{\left(1\right)}\left(3\right)\\ \vdots & \vdots & \ddots & \vdots \\ -z_{1}^{\left(1\right)}\left(n\right) & x_{2}^{\left(1\right)}\left(n\right) & \ldots & x_{N}^{\left(1\right)}\left(n\right) \end{array}\right)\in R^{\left(n-1\right)\;\times N}$$

Based on Remarks 1 and 2, it is obvious that GMC(1,N) is theoretically better than the classical GM(1,N) in three following aspects:

(i) In contrast to the GMC(1,N) model's structure, the conventional GM(1,N) cannot be transformed to GM(1,1).
(ii) The driving term $\sum_{i=2}^{N}\beta_{i}x_{i}^{\left(1\right)}\left(t\right)$ depends on t in GMC(1,N), yet this term is viewed as being fixed in the classical GM(1,N), which is obviously incorrect. Consequently, the cumulated forecast function of GM(1,N) is also incorrect.
(iii) GM(1,N) makes better sense when computing the parameters $A={\left[\alpha ,\;\beta_{2},\;.\;.\;.,\;\beta_{N},\;u\right]}^{T}$. The second to N^th^ column of B stems from integration in GMC(1,N), but in the conventional GM(1,N), they are treated as constants.

Even so, GMC(1,N) remains flawed in three respects:

(i) Parameterization mismatch: The least squares method is used to solve the difference equation in Eq. (6b) in order to estimate $A={\left[\alpha ,\;\beta_{2},\;.\;.\;.,\;\beta_{N},\;u\right]}^{T}$ from GMC(1,N). However, the first-order DE provided in Eq. (10) is used to create the time response function (see Eq (5)). Although the DE and the difference equation in Eq. (6b) approximate each other, they are fundamentally distinct. GMC(1,N) may become unstable as a result of this discrepancy in the estimation and application of the parameters.
(ii) Structural defects: GMC(1,N) is a causal model just like the conventional GM(1,N) [4], and therefore relies on various system factors. There are still some issues with structural rigidity in GMC(1,N). Though, its structure has undergone many modifications, most works have failed to address the linear impact of t on GMC(1,N)’s behaviour, which could explain why the model's predictive accuracy is poor.
(iii) Presence of noise in data: Finally, most studies have not taken into account the possibility that the input data may contain irrelevant information, which could also reduce the model's ability to predict outcomes accurately.

In view of the previous analysis, it follows that GMC(1,N) remains flawed due to inadequate parameter estimation and its structure is too simple to cope with real-world systems. These obvious defects urges us to develop a GMC(1,N) that overcomes them.

### The proposed optimal model

We briefly discussed the GMC(1,N) model in Section 2.1 and demonstrated that it is in fact an upgrade of the conventional GM(1,N). It suffers from several glaring flaws, though. In this section, a novel optimal discrete multivariate GMC model (abbreviated ODGMC(1,N)) is developed as a solution to these flaws, enhancing the precision and stability of the GMC(1,N) model while also addressing their causes.

#### ODGMC(1,N) model

We start by introducing a new definition that is based on the concept examined by Ref. [6].

Definition 2 Let Eqs. (1) and (2) still serve as the definitions for $X_{1}^{\left(0\right)}$ and $X_{1}^{\left(0\right)},\;\left(i\;=\;2,3,\;.\;.\;.,\;N\right)$. Then, considering the 1-AGO sequences $X_{i}^{\left(1\right)},\;\left(i\;=\;1,2,\;.\;.\;.,\;N\right)$ as in Eq (3). So:

(14) $$\frac{{dx}_{1}^{\left(1\right)}\left(t\right)}{dt}+{\alpha x}_{1}^{\left(1\right)}\left(t\right)=\sum_{i=2}^{N}\beta_{i}x_{i}^{\left(1\right)}\left(t\right)+\left(t-1\right)\lambda_{1}+\lambda_{2}$$

Eq. (14) is known as the optimized GMC(1,N) model's image equation. The system's development coefficient is −α, the system's driving term is $\sum_{i=2}^{N}\beta_{i}x_{i}^{\left(1\right)}\left(t\right)$ with driving coefficients $\beta_{i}$, whereas $\left(t-1\right)\;\lambda_{1}$ and $\lambda_{2}$ respectively represent the linear correction and model's parameter.

To improve the structure of GMC(1,N) in comparison, an additional linear adjustment term $\left(t-1\right)\lambda_{1}$ has been added in the RHS of Eq. (5). Many works on grey multivariate models (for example: Zeng et al. [[6], [7]]) have come to the conclusion that this linear adjustment factor is crucial and can easily change the degree to which $X_{1}^{\left(0\right)}$ and $X_{1}^{\left(0\right)},\;\left(i\;=\;2,3,\;.\;.\;.,\;N\right)$ are related. However, in these earlier studies, the DE is not used; instead, the linear correction factor is included right into the difference equation. Moreover, if we apply the same methodology used to derive the cumulated forecast function of GMC(1,N), we get an expression similar to Eq. (10), with the exception that f(τ) becomes:

$$f\left(\tau \right)=\sum_{i=2}^{N}\beta_{i}x_{i}^{\left(1\right)}\left(t\right)+\left(t-1\right)\lambda_{1}+\lambda_{2}$$

As a result, the parameter estimation mismatch flaw mentioned in Section 2.1 continues to exist. Fortunately, this mismatch issue can be eliminated by employing the discrete grey model methodology.

The novel ODGMC(1,N) outlined in the next subsections, is founded on optimized GMC(1,N) model's image equation described in Definition 2. When we integrate both sides of Eq. (14) in the range [k,k+1], we get:

(15) $$\int_{k}^{k+1}{dx}_{1}^{\left(1\right)}\left(t\right)+\int_{k}^{k+1}{\alpha x}_{1}^{\left(1\right)}\left(t\right)dt=\sum_{i=2}^{N}\beta_{i}\int_{k}^{k+1}x_{i}^{\left(1\right)}\left(t\right)dt+\int_{k}^{k+1}\left(t-1\right)\lambda_{1}dt+\int_{k}^{k+1}\lambda_{2}dt$$

By recalling that: $x_{1}^{\left(1\right)}\left(k+1\right)-x_{1}^{\left(1\right)}\left(k\right)=x_{1}^{\left(0\right)}\left(k+1\right)$, Eq. (15) can be written as:

$$x_{1}^{\left(0\right)}\left(k+1\right)+\alpha \int_{k}^{k+1}x_{1}^{\left(1\right)}\left(t\right)dt=\sum_{i=2}^{N}\beta_{i}\int_{k}^{k+1}x_{i}^{\left(1\right)}\left(t\right)dt+\left(k-\frac{1}{2}\right)\lambda_{1}+\lambda_{2}$$

In order to integrate the undefined functions, we can use the trapezoidal formula as follows:

(16) ∫kk+1xi(1)(t)dt=(xi(1)(k+1)+xi(1)(k))2Δ=zi(1)(k+1)

Applying this formula to both unknown terms in Eq. (15), we get:

(17) $$x_{1}^{\left(0\right)}\left(k+1\right)+{\alpha z}_{1}^{\left(1\right)}\left(k+1\right)=\sum_{i=2}^{N}\beta_{i}z_{i}^{\left(1\right)}\left(k+1\right)+\left(k-\frac{1}{2}\right)\lambda_{1}+\lambda_{2}$$

For the LHS of Eq. (17), we have:

(18) $$\left\{ \begin{array}{c} x_{1}^{\left(0\right)}\left(k+1\right)+{\alpha z}_{1}^{\left(1\right)}\left(k+1\right)=x_{1}^{\left(1\right)}\left(k+1\right)-x_{1}^{\left(1\right)}\left(k\right)+\frac{\alpha }{2}\left(x_{1}^{\left(1\right)}\left(k+1\right)+x_{1}^{\left(1\right)}\left(k\right)\right)\;\\ \;\;\;=\left(1+\frac{\alpha }{2}\right)x_{1}^{\left(1\right)}\left(k+1\right)-\left(1-\frac{\alpha }{2}\right)x_{1}^{\left(1\right)}\left(k\right) \end{array}\right.$$

Substituting Eq. (18) into Eq. (17) and simplifying, we have:

(19) $$x_{1}^{\left(1\right)}\left(k+1\right)=\varphi_{1}x_{1}^{\left(1\right)}\left(k\right)+\sum_{i=2}^{N}\varphi_{i}z_{i}^{\left(1\right)}\left(k+1\right)+\mu_{1}k+\mu_{2}$$

where: $\varphi_{1}=\frac{\left(1-\frac{\alpha }{2}\right)}{\left(1+\frac{\alpha }{2}\right)}$; $\varphi_{i}=\frac{\beta_{i}}{\left(1+\frac{\alpha }{2}\right)},\;\left(i=2,3,\ldots ,N\right)$; $\mu_{1}=\frac{\lambda_{1}}{\left(1+\frac{\alpha }{2}\right)}$; $\mu_{2}=\frac{\lambda_{2}-\frac{\lambda_{1}}{2}}{\left(1+\frac{\alpha }{2}\right)}$

Eq. (19) is then called the optimised discrete multivariate GMC model.

#### Parameterization of the optimised discrete GMC(1,N) model

From Eq. (19), it comes out that N+2 parameters given by the matrix $A={\left[\varphi_{1},\;\varphi_{2},\;.\;.\;.,\;\varphi_{N},\;\mu_{1},\mu_{2}\right]}^{T}$ need to be obtained by finding the solution of the linear set of equations in Eq. (20):

(20) BA=Y

where,

(21) $$\left\{ \begin{array}{c} B=\left(\begin{array}{cccccc} x_{1}^{\left(1\right)}\left(1\right) & z_{2}^{\left(1\right)}\left(2\right) & \ldots & z_{N}^{\left(1\right)}\left(2\right) & 1 & 1\\ x_{1}^{\left(1\right)}\left(2\right) & z_{2}^{\left(1\right)}\left(3\right) & \ldots & z_{N}^{\left(1\right)}\left(3\right) & 2 & 1\\ \vdots & \vdots & \ddots & \vdots & \vdots & \vdots \\ x_{1}^{\left(1\right)}\left(n-1\right) & z_{2}^{\left(1\right)}\left(n\right) & \ldots & z_{N}^{\left(1\right)}\left(n\right) & n-1 & 1 \end{array}\right)\in R^{\left(n-1\right)\;\times \;\left(N+2\right)}\\ Y=\left(\begin{array}{c} x_{1}^{\left(1\right)}\left(2\right)\\ x_{1}^{\left(1\right)}\left(3\right)\\ \vdots \\ x_{1}^{\left(1\right)}\left(n\right) \end{array}\right)\in R^{\left(n-1\right)\times 1} \end{array}\right.$$

#### Evaluation of the predicted accumulated and original series

The following theorem describes the application of the recursive technique (similar to that of discrete grey models [8,9]) in order to determine the ODGMC(1,N) model's cumulated forecast function:

Theorem: The cumulated forecast function of ODGMC(1,N), as specified in Eq. (19), is as follows under the initial condition ${\hat{x}}_{1}^{\left(1\right)}\left(t=1\right)=x_{1}^{\left(0\right)}\left(1\right)$:

(22) $${\hat{x}}_{1}^{\left(1\right)}\left(t+1\right)=\varphi_{1}^{t}x_{1}^{\left(0\right)}\left(t\right)+\sum_{j=1}^{t}\varphi_{1}^{t-j}\left(\sum_{i=2}^{N}\varphi_{i}z_{i}^{\left(1\right)}\left(j+1\right)+\mu_{1}j+\mu_{2}\right),\;t\geq 1$$

The appendix shows the proof. This Theorem demonstrates that Eq. (22) can be used to evaluate the 1-AGO series ${\hat{x}}_{1}^{\left(1\right)}$. As a result, the inverse-AGO can be used to determine the forecasted series ${\hat{x}}_{1}^{\left(0\right)}$ as follows:

$${\hat{x}}_{1}^{\left(0\right)}\left(t+1\right)={\hat{x}}_{1}^{\left(1\right)}\left(t+1\right)-{\hat{x}}_{1}^{\left(1\right)}\left(t\right),\;t\geq 1$$

#### Data filtration based on wavelet transform

ODGMC(1,N) may be corrupted by noise or useless information in the raw data. Wavelet transform (WT) can be used to clean this noise [10]. A wavelet is a mathematical function that separates various scale components from a continuous-time signal. Basically, WT is a band-pass filter with its bandwidth scaled to half at each level [11]. The scaling function makes sure that the entire spectrum is considered by filtering away the transform's lowest level. Eq. (23) applies when a signal x(t) is continuous:

(23) $$CWT_{\Psi }\left(\eta ,\vartheta \right)=\frac{1}{\sqrt{\left|\eta \right|}}\int_{-\infty }^{+\infty }x\left(t\right)\Psi^{*}\left(\frac{t-\vartheta }{\eta }\right)dt$$

where the scale and translation parameters, are denoted by η and ϑ (η,ϑ∈R) respectively. Eq. (23) is called the continuous wavelet transform (CWT).

With a discrete signal $x_{j}$, a discrete WT (DWT) is calculated using Eq. (24):

(24) $$DWT_{x}\left(p,q\right)=\frac{1}{\sqrt{2^{p}}}\sum_{j}x_{j}\Psi^{*}\left(\frac{j-q}{2^{p}}\right)$$

where q=1,2,…N and p are the sampling time and scale factor respectively. N is the number of samples. The most crucial element of the signal is its low order component. The signal's identity is clarified in this component. The signal's high order component, on the other hand, is a representation of the signal's specifics.

## Applications and numerical simulation

### Data selection

Data used in this method paper cover the period 2000–2019 and were collected from IEA and World Bank's development indicators (WDI). Specifically, dataset on electricity consumption is from IEA (*https://www.iea.org/*). In contrast, datasets on GDP per capita, household expenses and population size come from WDI (*https://databank.worldbank.org*) and are confirmed by the National Institute of Statistics (*https://ins-cameroun.cm/*).

### Performance evaluation

The reliability and forecast precision are evaluated using Root Mean Square Error (RMSE), Mean Square Error (MSE), Mean Absolute Percentage Error (MAPE) and Absolute Percentage Error (APE).

MAPE and APE disclose the models’ predictive accuracy. MAPE in particular is a performance metric that compares the accuracy of forecasts based on relative errors to prevent positive and negative errors from mutually annulling. Threshold values of MAPE are given in Table 1. The formulae for calculating APE and MAPE are defined by Eqs. (25) and (26):

(25) $$\mathrm{APE}=\left|\frac{{\hat{x}}_{1}^{\left(0\right)}\left(t\right)-x_{1}^{\left(0\right)}\left(t\right)}{x_{1}^{\left(0\right)}\left(t\right)}\right|\times \;100\%$$

(26) $$\mathrm{MAPE}=\frac{1}{N_{s}}\sum_{t=1}^{N_{s}}\left|\frac{{\hat{x}}_{1}^{\left(0\right)}\left(t\right)-x_{1}^{\left(0\right)}\left(t\right)}{x_{1}^{\left(0\right)}\left(t\right)}\right|\times \;100\%$$

**Table 1:** Thresholds for MAPE [12].

MAPE(%)	Accuracy level	MAPE(%)	Accuracy level
]0;5]	Class I: Extremely high precision	]10;20]	Class III: Average precision
]5;10]	Class II: Good precision	]20;+∞[	Class IV: Low precision

MSE (defined in Eq. (27)) is very often considered as a loss function. It is calculated by adding the square of the difference between real $x_{1}^{\left(0\right)}\left(t\right)$ and forecasted electricity consumption ${\hat{x}}_{1}^{\left(0\right)}\left(t\right)$, over all the data points and dividing the result by number of data points.

(27) $$MSE=\frac{1}{N_{s}}\sum_{t=1}^{N_{s}}{\left({\hat{x}}_{1}^{\left(0\right)}\left(t\right)-x_{1}^{\left(0\right)}\left(t\right)\right)}^{2}$$

RMSE (Eq. (28)) is the square root of MSE. RMSE acts much like the MSE except that it is prone to inflate significant deviations [13], and this may be useful when comparing competing models.

(28) $$RMSE=\sqrt{MSE}$$

The best performing model in indicated by a score of RMSE, MSE, MAPE and APE that is closest to zero. However, we focus more on MAPE because it is a metric that comes up very often in forecasting studies. MAPE is usually expressed as a percentage error making it easy to grasp and compare the accuracy of a model across data sets and case studies.

### Forecasting annual electricity consumption

**Fig. 1:**
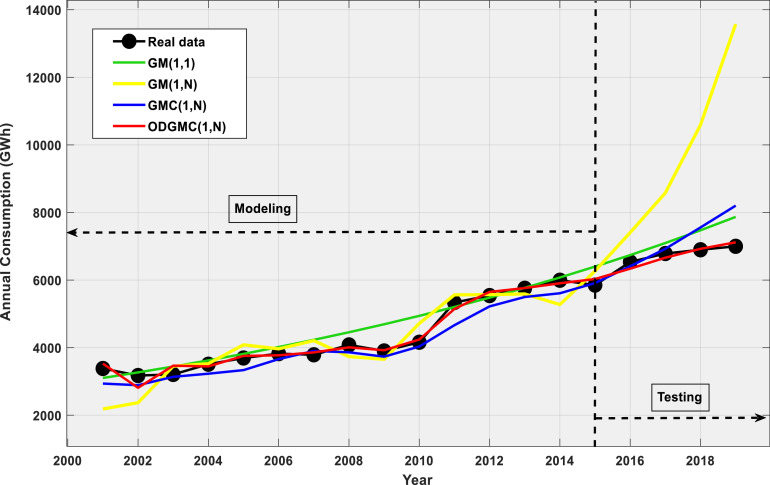
Fitting curves of ODGMC(1,N) and competing models.

Simulations were performed on a Spyder 64 bit IDE on i7 personal computer with 16Go of RAM. Datasets for the period 2000–2015 served as modelling data while those for the period 2016–2019 were used as validation sets. During simulations, validation data were hidden to prevent any leakage in order to verify whether the models were overfitting or underfitting. Simulation outcomes are displayed on Table 2, whereas Fig. 1 provides a visual representation of these results. There is evidence of the failure of the conventional GM(1,1) to accurately capture the system's evolution law.

**Table 2:** Simulation results for the modelling and validation phases.

Training									
Year	Real consumption	GM(1,1)	APE	GM(1,N)	APE	GMC(1,N)	APE	ODGMC(1,N)	APE
2000	3541.0								
2001	3382.5	3102.0	8.3	2183.6	35.5	2936.2	13.2	3525.7	4.2
2002	3174.2	3266.6	2.9	2369.7	25.4	2888.2	9.0	2811.3	11.4
2003	3206.7	3440.0	7.3	3449.5	7.6	3136.0	2.2	3465.7	8.1
2004	3508.8	3622.6	3.2	3526.8	0.5	3228.6	7.9	3444.3	1.8
2005	3693.2	3814.9	3.3	4083.9	10.6	3334.7	9.7	3755.3	1.7
2006	3822.1	4017.4	5.1	3963.3	3.7	3660.3	4.2	3772.8	1.3
2007	3788.1	4230.7	11.7	4206.9	11.1	3901.9	3.0	3859.3	1.9
2008	4080.2	4455.2	9.2	3734.1	8.5	3861.8	5.4	4008.4	1.8
2009	3901.5	4691.7	20.3	3654.7	6.3	3732.6	4.3	3922.0	0.5
2010	4159.6	4940.8	18.8	4715.2	13.4	4032.9	3.0	4240.4	1.9
2011	5336.0	5203.0	2.5	5561.8	4.2	4664.3	12.6	5143.7	3.6
2012	5541.0	5479.2	1.1	5568.4	0.5	5220.6	5.8	5648.9	2.0
2013	5757.0	5770.0	0.2	5589.4	2.9	5498.4	4.5	5763.3	0.1
2014	5994.8	6076.3	1.4	5274.6	12	5609.0	6.4	5904.5	1.5
2015	5851.0	6398.8	9.4	6267.2	7.1	5910.7	1.0	6030.6	3.1
	**MAPE**	6.97%		9.94%		6.16%		2.99%	
	**MSE**	526,801.26		250,203.67		93,174.31		22,395.58	
	**RMSE**	725.81		500.20		305.24		149.65	
Testing									
2016	6536.5	6738.5	3.1	7406.5	13.3	6410.0	1.9	6336.9	3.1
2017	6785.2	7096.2	4.6	8580.6	26.5	6925.4	2.1	6661.6	1.8
2018	6896.6	7472.9	8.4	10,593.5	53.6	7557.6	9.6	6924.7	0.4
2019	6998.4	7869.6	12.5	13,573.0	94.0	8202.0	17.2	7116.6	1.7
	**MAPE**	7.12%		46.83%		7.70%		1.74%	
	**MSE**	307,164.11		15,218,542.40		480,272.71		17,465.21	
	**RMSE**	554.22		3901.10		693.02		132.16

GM(1,N) predictions (yellow curve) fit moderately well in the modelling phase (Table 2) but completely deviates in the validation phase, meaning it is overfitting. Of all alternative models, only the GMC(1,N) manages to compete with the new ODGMC(1,N) model. It can be seen from Fig. 1 that the new ODGMC(1,N) manages to correctly track the system's evolution in both the modelling and validation phases.

**Fig. 2:**
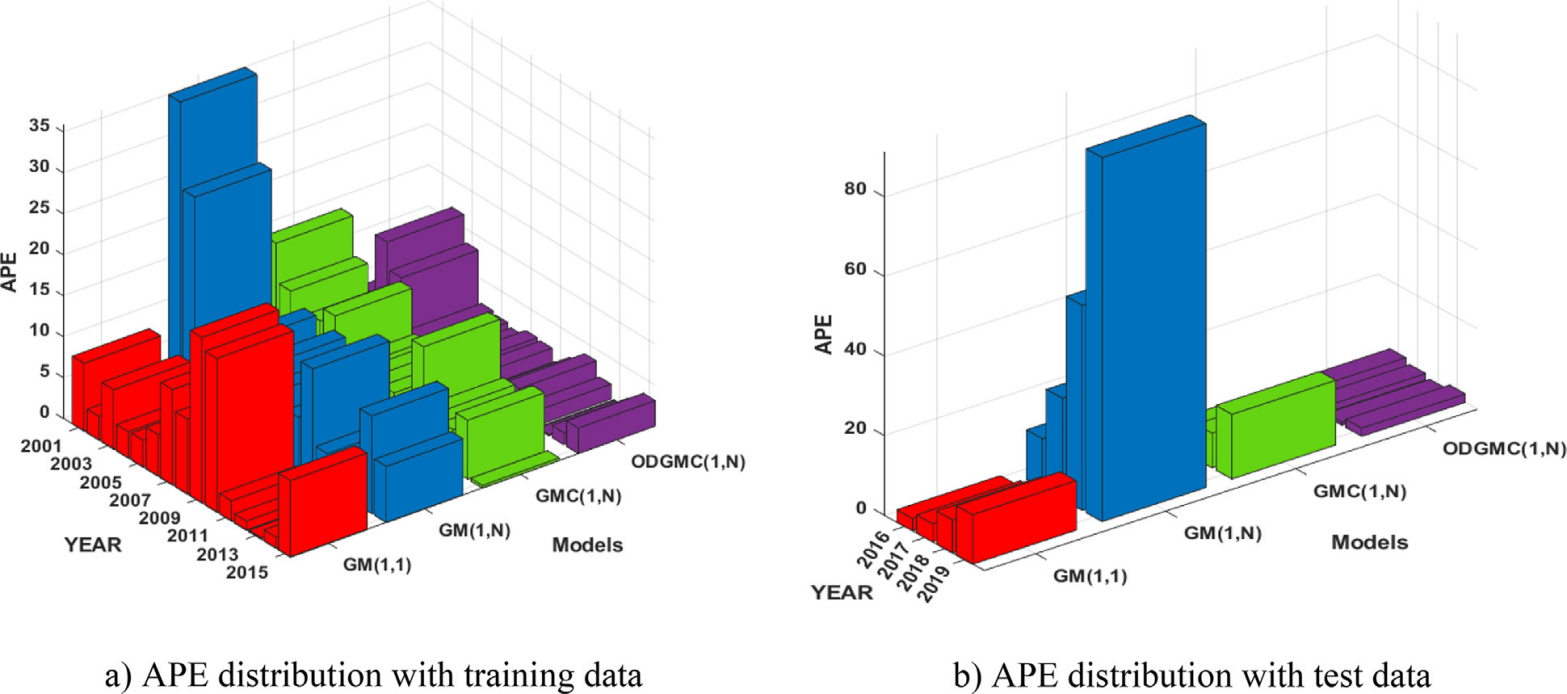
APE distribution of simulations.

APE distribution (see Fig. 2) for the training and validation phases shows that the residuals of ODGMC(1,N) model are much more smaller than those of GM(1,1), GM(1,N) and GMC(1,N). So, this also reiterates the superiority of ODGMC(1,N) model. MAPE criteria in particular, with statistics of 2.74% and 1.99% in the modelling and validation phase respectively, demonstrates that ODGMC(1,N) is a class I model and can compete with high accuracy models.

## Conclusion and future works

A new and innovative approach is developed in this paper with the aim to improve the predictive accuracy of GMC(1,N). More specifically, an optimal version abbreviated ODGMC(1,N) is formulated and implemented in this work. This forecasting model is based on the concepts of discrete GMs and on wavelet data filtering techniques. The novel ODGMC(1,N) corrects two flaws in the modelling structure of GMC(1,N) and has the ability to excavate the links that exist between a series and its drivers. The structure of the novel model and its parameterisation are also carefully considered. The prediction of Cameroon's annual electricity demand is used to demonstrate that ODGMC(1,N) has a significantly higher forecast precision and improved stability than competing models. Moreover, ODGMC(1,N) succeeds in achieving this with a limited number of explanatory variables. The forecasting outcomes confirm that ODGMC(1,N) succeeds in predicting the demand for electricity in Cameroon with MAPE, RMSE, and MSE of 1.74%, 132.16, and 17,465.21, respectively, categorizing it as a very high precision model. These interesting results are due to:

• The stability of ODGMC(1,N) resulting from a good adequacy between parameters estimation and their implementation.
• The addition of a term that takes into account the linear impact of t on the model's performance.
• The removal of irrelevant information from input data by wavelet transform filtration.

One limit of the proposed model is that it cannot fully extract information from seasonal series that exhibits sharp fluctuations. This is because the WT will perceive these variations as noise when they are not, and once WT has damped down what it takes to be a disturbance, the system's information will be lost resulting in poor forecasts.

## CRediT authorship contribution statement

**Flavian Emmanuel Sapnken:** Conceptualization, Methodology, Software, Writing – original draft. **Marius Tony Kibong:** Data curation, Visualization, Investigation. **Jean Gaston Tamba:** Supervision, Validation, Writing – review & editing.

## Declaration of Competing Interest

The authors declare that they have no known competing financial interests or personal relationships that could have appeared to influence the work reported in this paper.

## Appendix. Proof of theorem leading to Eq. (22)

Proof: Let $\sum_{i=2}^{N}\varphi_{i}z_{i}^{\left(1\right)}\left(t+1\right)+\mu_{1}t+\mu_{2}=w\left(t\right)$ be an auxiliary function (that we introduce for convenience).

According to Eq. (19), the cumulated forecast function of ODGMC(1,N) can be determine as follows:

$${\hat{x}}_{1}^{\left(1\right)}\left(t+1\right)=\varphi_{1}{\hat{x}}_{1}^{\left(1\right)}\left(t\right)+w\left(t\right)=\varphi_{1}\left(\varphi_{1}{\hat{x}}_{1}^{\left(1\right)}\left(t-1\right)+w\left(t-1\right)\right)+w\left(t\right)=\varphi_{1}^{2}{\hat{x}}_{1}^{\left(1\right)}\left(t-1\right)+\varphi_{1}w\left(t-1\right)+w\left(t\right)=\cdots =\varphi_{1}^{t}{\hat{x}}_{1}^{\left(1\right)}\left(1\right)+\varphi_{1}^{t-1}w\left(1\right)+\varphi_{1}^{t-2}w\left(2\right)+\cdots +\varphi_{1}w\left(t-1\right)+w\left(t\right)$$

Under the initial condition ${\hat{x}}_{1}^{\left(1\right)}\left(t=1\right)=x_{1}^{\left(0\right)}\left(1\right)$, we have:

$${\hat{x}}_{1}^{\left(1\right)}\left(t+1\right)=\varphi_{1}^{t}x_{1}^{\left(0\right)}\left(t\right)+\sum_{j=1}^{t}w\left(j\right)\varphi_{1}^{t-j}.$$

Substituting $\sum_{i=2}^{N}\varphi_{i}z_{i}^{\left(1\right)}\left(t+1\right)+\mu_{1}t+\mu_{2}=w\left(t\right)$ in the previous expression immediately results in the given time response function defined by Eq. (22). This completes the proof.

## Footnotes

1 Time response function is sometimes called the cumulated forecast function [5].

2 Other high precision numerical integrals, such as the Gaussian formula, could also be used to calculate the convolution integral in Eq. (10) [14].
